# Is long time to reimplantation a risk factor for reinfection in two-stage revision for periprosthetic infection? A systematic review of the literature

**DOI:** 10.3389/fsurg.2023.1113006

**Published:** 2023-02-17

**Authors:** Jan Puetzler, Martin Schulze, Georg Gosheger, Jan Schwarze, Burkhard Moellenbeck, Christoph Theil

**Affiliations:** Department of Orthopaedics and Tumor Orthopaedics, University Hospital Muenster, Muenster, Germany

**Keywords:** periprosthetic joint infection, two-stage exchange, revision arthroplasty, time to reimplantation, spacer interval, TKA, THA

## Abstract

The two-stage revision arthroplasty is a common treatment option for chronic periprosthetic infection (PJI). The time to reimplantation (TTR) reported in the literature varies substantially from a few days to several hundred days. It is hypothesized that longer TTR could be associated with worse infection control after second stage. A systematic literature search was performed according to Preferred Reporting items for Systematic Reviews and Meta-Analyses (PRISMA) guidelines, in Pubmed, Cochrane Library and Web of Science Core Collection in clinical studies published until January 2023. Eleven studies investigating TTR as a potential risk factor for reinfection met the inclusion criteria (ten retrospective and one prospective study, published 2012–2022). Study design and outcome measures differed notably. The cutoff points above which TTR was regarded as “long” ranged from 4 to 18 weeks. No study observed a benefit for long TTR. In all studies, similar or even better infection control was observed for short TTR. The optimal TTR, however, is not yet defined. Larger clinical studies with homogeneous patient populations and adjustment for confounding factors are needed.

## Introduction

Periprosthetic joint infection (PJI) is a feared complication in orthopedic surgery that requires complex surgical procedures and long systemic treatments aiming at infection control. This is an enormous burden for affected patients and results in high costs for the health care system (1). The infection risk after primary total hip or knee arthroplasty is 1%–2% (2), but the risk for recurrence of infection can reach up to 50% in complex cases after multiple revisions (3–6). The current gold standard for chronic PJI is the two-stage revision arthroplasty (7, 8). A temporary polymethyl methacrylate (PMMA) spacer fills the debrided joint space, bridges bony defects, stabilizes the joint and ideally maintains the length of the extremity. In addition, local anti-infective substances mixed in the PMMA are released into the surrounding, reaching very high local concentrations, with little risk of systemic side effects (9). However, surgeons in clinical practice are confronted with the issue of timing second stage reimplantation surgery. From a patient's perspective, a short interval appears preferable to regain the ability to use the affected limb in everyday life. Yet, various factors such as comorbidities, clinical examination, laboratory results and organizational factors influence the time to reimplantation (TTR) (10). A widely adopted classification by Trampuz and Zimmerli defines intervals of two to four weeks (short interval) and six to eight weeks (long interval) until reimplantation (11). Other authors suggest four to six weeks (12), or nine weeks between the stages (13). However, spacer intervals reported in clinical studies often exceeded the time periods of guideline recommendations. They range from a few days to several hundred days, but mostly an average interval around 80 to 100 days is reported (4, 7, 14–22),. This heterogeneity in clinical practice indicates that an optimal interval period between the stages, has not been conclusively defined. In this study, we systematically searched the literature for studies that described two-stage revision arthroplasty of the hip and knee and analyzed the outcome “reinfection” in relation to the TTR.

## Methods

The preferred reporting items for Systematic Reviews and Meta-Analyses (PRISMA) guidelines and the Cochrane Handbook for Systematic Reviews of Interventions were followed (23, 24).

### Data sources

Electronic searches were performed in the databases PubMed (including MEDLINE; 1970 to 2023), Cochrane Library (1970 to 2023), and Web of Science Core Collection (1970 to 2023) to identify relevant studies. For PubMed and the Cochrane Library, index terms (MeSH-terms) were included and combined with free text words to search in title, abstract, and keywords. We used four concepts (1. Arthroplasty, 2. Infection, 3. Treatment, 4. Humans). These four concepts were combined with the Boolean operator “AND”. The operator “NOT” was used to exclude case reports and reviews. The search was performed on January 1, 2023. The full search strategy is available in the Supplementary Material.

### Study selection

**Figure 1:**
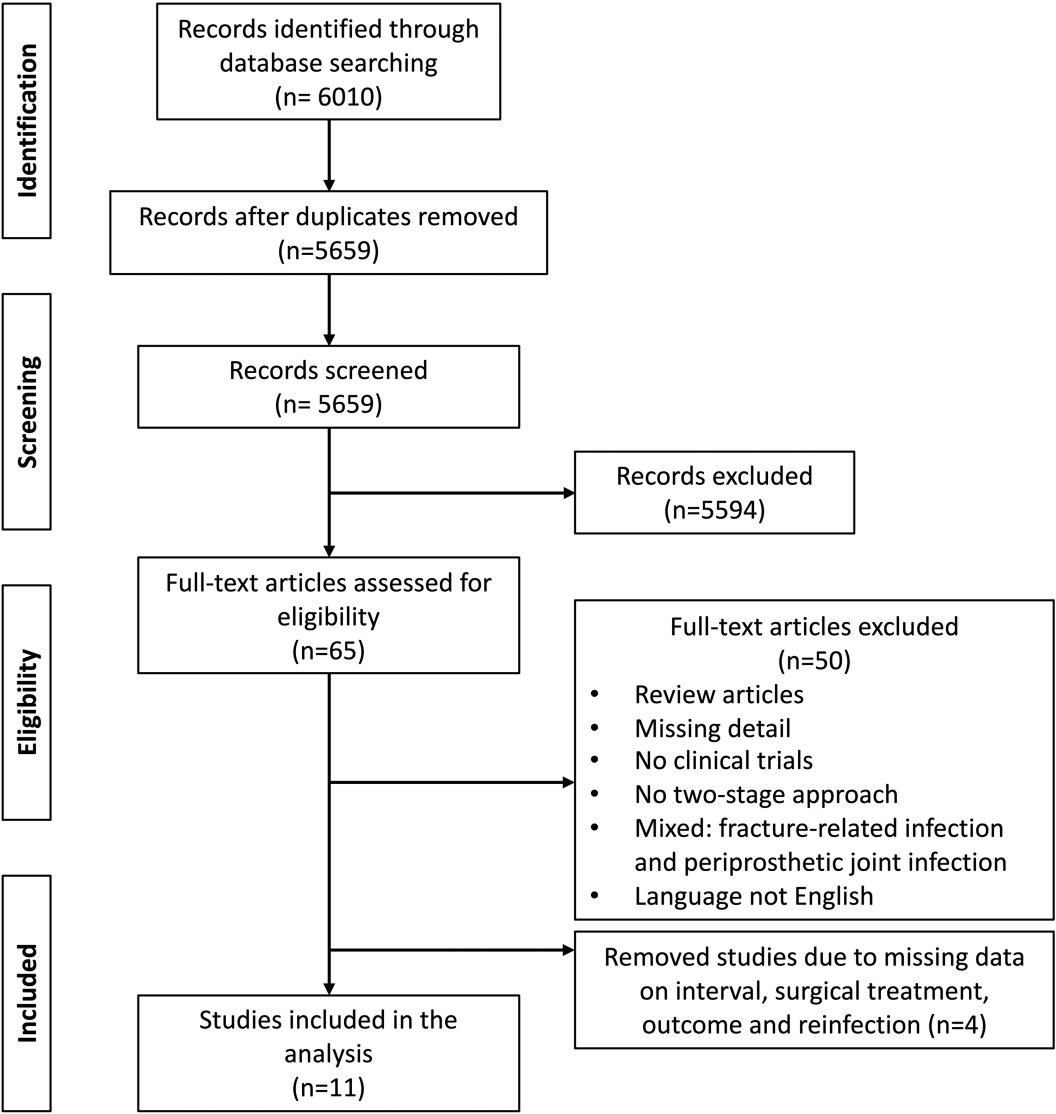
Preferred reporting items for systematic reviews and meta-analyses (PRISMA) flow diagram: eligibility assessment.

After identification of 6,010 publications, duplicates were removed and eligible studies were selected by the authors in three phases, resulting in eleven included studies (Figure 1). Eligibility criteria were set as follows: 1) population: Adult humans with chronic PJI of hip and knee, 2) intervention: treatment with completed two-stage revision arthroplasty 3) outcome: reinfection after the second stage; and 4) study design – retrospective cohort studies, prospective cohort studies and Randomized Controlled Trials (RCT). Technical notes were excluded. Only studies providing information on the time to reimplantation (TTR) after the first stage of a completed two-stage revision arthroplasty were included. We excluded the following studies: studies of paediatric patients; studies not involving endoprostheses of the hip and knee; treatment of septic arthritis of native joints, treatment of PJI with one-stage revision arthroplasty, DAIR procedure (Debridement, antibiotics, implant retention) or only partial removal of prosthesis components; studies that did not provide sufficient information on the surgery, experimental or animal studies; and studies written in languages other than English. After removal of duplicates, 5,659 titles and abstracts were screened. A total of 65 clinical studies evaluated the outcome of two-stage revision arthroplasty and reported on the time to reimplantation (TTR). The full-text analysis lead to the exclusion of 54 articles. Eleven studies met the inclusion criteria and were included in the analysis (Figure 1).

### Data analysis

A descriptive analysis was performed by comparing the risk of reinfection in the observation period after completed second stage, in relation to the time to reimplantation (TTR: time interval between first and second stage). In addition, potential sources for bias were identified.

## Results

### Study characteristics

The included studies and their main characteristics are summarized in Table 1. All were published between 2012 and 2022 and reported on a total of 1,552 patients treated between 1996 and 2019. Ten studies were retrospective, and one was a prospective cohort study.

**Table 1:** Overview of eleven studies assessing the association of the time to reimplantation (TTR) and the risk of reinfection after two-stage revision arthroplasty.

Study	Year	Study design	Population	Outcome	Results	Limitations and potential bias
Kubista *et al.* (25)	2012	Retrospective Study period: 1998-2006 Follow-up interval: 6-2,853 days	116 patients with knee PJI, completed two-stage exchange	1. TTR in a cohort with reinfection compared to controls without reinfection 2. TTR as continuous variable regarding outcome reinfection.	1. Median TTR 66 days (reinfection) vs. 61 days (no reinfection) 2. Additional 30 days TTR: Hazard ratio: 1.14 (*p* = 0.03)	• Retrospective • 26 patients had additional revision and/or spacer exchanges between stages (not excluded). • No information on spacer type
Sabry *et al.* (17)	2014	Retrospective Study period: 1996-2010 Follow-up interval: mean: 1,215 days (range, 59–4,202 days	314 cases of knee PJI, completed two-stage exchange	TTR in a cohort with reinfection compared to controls without reinfection	Median TTR 124 days (reinfection) vs. 96 days (no reinfection) (*p* = 0.015)	• Retrospective • different spacer types were used • no adjustment for potential confounding factors (comorbidities) • cases with additional revision and/or spacer exchanges between stages were not excluded (no exact number)
Winkler *et al.* (14)	2019	Prospective, quasi randomized to short and long interval in two different time periods Study period: Jan-Dec. 2013 Follow-up interval: Minimum follow-up: n.a., Mean follow-up: 3.3 years	38 patients with hip or knee PJI, completed two-stage revision TKA. Reimplantation within 4 weeks (short interval, *n* = 19) or ≥4 weeks (long interval, *n* = 19)	Reinfection rate	One patient with reinfection in the long interval group, no patients with reinfection in the short interval group	• Small cohort • no information on missing data/drop-out • no adjustment for comorbidities
Akgün *et al.* (26)	2019	Retrospective Study period: 2013-2015 Follow-up interval: Minimum follow-up: 2 years, Mean follow-up: 33.1 months	84 patients with two-stage revision THA Reimplantation within 6 weeks (short interval, *n* = 18) or > 6 weeks (long interval, *n* = 66)	1. Kaplan-Meier Infection free survival 2. Reinfection rate	1. All patients: infection free survival: 3 years 89.3% (95% CI, 80% to 94%) with 30 patients at risk Reinfection: 0 patients in short interval group vs. 9 patients in long interval group (*p* = 0.02)	• Retrospective • Small cohort • 13 patients had additional revision and/or spacer exchanges between stages (not excluded).
Hipfl *et al.* (27)	2019	Retrospective Study period: 2014-2015 Follow-up interval: Minimum follow-up: 2 years, Mean follow-up: 41 months	97 patients with knee PJI, completed two-stage exchange using static spacers	Time to reimplantation (TTR) in a cohort with reinfection (*n* = 15) compared to controls without reinfection (*n* = 82)	Median TTR 71 days (reinfection) vs. 64 days (no reinfection) (*p* = 0.393)	• Retrospective • Small cohort • 9 patients had additional revision and/or spacer exchanges between stages (not excluded).
Tan *et al.* (28)	2018	Retrospective Study period: 2000-2014 Follow-up interval: Minimum follow-up: 1 year, Mean follow-up: n.a.	409 patients with knee PJI, completed two-stage exchange, Patients in need of revision surgeries between stages were excluded.	1. Reinfection rate (association with duration of antibiotic holiday and TTR) 2. Time to reimplantation (TTR) in a cohort with reinfection compared to controls without reinfection	1. association of reinfection risk with TTR (but not with antibiotic holiday). Notable increase of reinfection risk for TTR >100 days. 2. Median TTR 113 days (reinfection) vs. 88 days (no reinfection) (*p* = 0.037)	• Retrospective data over 14 years from two institutions with varying surgeons and protocols • different spacer types were used • only graphical presentation of increased risk for TTR >100 days. • No adjustment for confounding factors (regarding TTR)
Sigmund *et al.* (29)	2019	Retrospective Study period: 2006-2014 Follow-up interval: Minimum follow-up: 1 year, Mean follow-up: 3.6 years	93 patients with hip PJI; completed two-stage exchange with resection arthroplasty (no spacer) Reimplantation within 10 weeks (short interval, *n* = 49) or > 10 weeks (long interval, *n* = 44)	Kaplan-Meier Infection free survival	Infection free survival at 12 months: 94% (short) and 91% (long); at 24 months: 94% (short) and 86% (long) (log-rank test, *p* = 0.223)	• Retrospective • no adjustment for comorbidities (BMI sign. different)) • additional revision and/or spacer exchanges between stages were not excluded. • small cohort (Reinfection in only nine patients)
Vielgut *et al.* (30)	2015	Retrospective Study period: 2005-2010 Follow-up interval: Minimum follow-up: n.a., Mean follow-up: 20.5 months	76 patients with acute and chronic hip PJI with intended two-stage exchange.	optimal TTR cutoff with maximally selected log-rank statistic	Calculated cutoff-points: Increased rate of reinfection before 4 weeks (5/5) and after 11 weeks (11/23). Patients with TTR 4-11 weeks had lowest rate of reinfection (36/40) (*p* = 0.014)	• Retrospective • 13 patients had additional revision and/or spacer exchanges between stages and eight patients did not reach second stage reimplantation • No difference was made between acute and chronic infections • No adjustment for confounding factors • Small cohort. Only five patients in the group with reimplantation within 4 weeks
Vielgut *et al.* (31)	2021	Retrospective Study period: n.a. Follow-up interval: Minimum follow-up: n.a., Mean follow-up: 24.5 months	77 patients with knee PJI, completed two-stage exchange using static spacers	1. Kaplan-Meier Infection free survival and optimal TTR with maximally selected log-rank statistic 2. TTR as continuous variable regarding outcome reinfection.	1. Calculated cutoff-point: 83 days (12 weeks). Kaplan Meier estimate of reinfection rate at five years was 13.7% for shorter TTR and 46,9% for longer TTR. 2. Hazard ratio was 6.1 (95% CI: 1.6-22.9, *p* = 0.007) if TTR was longer than 12 weeks	• Retrospective • 17 patients had additional revision and/or spacer exchanges between stages (not excluded). • No adjustment for confounding factors • Small cohort • patients had additional revision and/or spacer exchanges between stages
Borsinger *et al.* (32)	2022	Retrospective Study period: 2011-2018 Follow-up interval: Minimum follow-up: 2 years Mean follow-up: n.a.	90 patients with hip or knee PJI, completed two-stage exchange (after excluding 11 patients with revision between stages)	Treatment failure at two years (reoperation or death)	If TTR was >18 weeks the risk for reinfection was significantly increased (Odds ratio: 4.12, CI 95%: 1.18-15.37, *p* = 0.029) Adjusted for health status (ASA Score and number of prior revisions)	• Retrospective • cutoff-point (18 weeks) was chosen arbitrarily • different spacer types were used • small cohort
Hartman *et al.* (33)	2022	Retrospective Study period: 2008-2019 Follow-up interval: Minimum follow-up: 2 years, Mean follow-up: 3.4 years	158 patients with hip and knee PJI; with completed two-stage exchange and articulating spacers if possible	1. Reinfection rate 2. TTR in group with reinfection compared to group without reinfection	1. Reinfection rate: 19.6% (31/158) 2. Median TTR 141 days (reinfection) vs. 109 days (no reinfection) (*p* = 0.055)	• retrospective • no information on revisions between stages • no adjustment for comorbidities • no adjustment for multiplicity, no information on missing data/drop-out

ASA Score: American Society of Anesthesiologists Score; TTR: time to reimplantation; n.a.: not applicable/not reported.

### Reinfection after two-stage revision arthroplasty and time to reimplantation (TTR)

Kubista et al*.* compared risk factors from 58 patients with reinfections after two-stage exchange of total knee arthroplasty (TKA) with 58 patients they randomly selected from a cohort without reinfection (25). The median TTR in their study was 66 days in the reinfected group and 61 days in control group. They also considered TTR as a continuous variable and calculated a hazard ratio for additional 30 days TTR of 1.14 (*p* = 0.03). However, they included a relevant proportion of patients that required additional revision and spacer exchanges before reimplantation (*n* = 26, 22%; *n* = 17 in the reinfected group and *n* = 9 in the group without reinfection *p* = 0.01). This could be a confounding factor as these revisions likely prolonged the TTR and are considered themselves a risk factor for reinfection (34–36).

Sabry et al*.* identified TTR as an independent risk factor among 314 patients with knee PJI undergoing a two-stage exchange with a median of 124 days until reimplantation in the reinfected group vs. 96 days in the group without reinfection (*p* = 0.015) (17). Again, patients requiring a spacer exchange in between the stages were not excluded from the analysis.

Winkler et al*.* published a small series of patients with hip and knee PJI receiving reimplantation either within four weeks (*n* = 19) or thereafter (*n* = 19) (14). Cases with difficult to treat microorganisms and patients with critical soft tissues were excluded. On average the short interval group had a mean of 17.9 days compared to 63 days in the long interval group. Only one reinfection was observed in this cohort in the long interval group, therefore the authors suggested that the shorter interval might at least achieve similar infection control compared to longer intervals.

Akgün et al*.* from the same group published a cohort of 18 patients with hip PJI in 2019 with an interval of less than 6 weeks and 66 patients with a longer interval (26). Mean time interval of all patients between stages was reported 60.9 days (8.7 weeks, range: 1–25). Girdlestone resection arthroplasty without the use of cement spacers was the preferred treatment approach. Thirteen patients required revision surgery between the stages due to infection persistence and were kept in the analysis. Reinfection was observed in none of the patients in the shorter interval group and in nine patients in the longer interval group, however this difference was not significant.

Hipfl et al*.* reviewed 97 cases of knee PJI with static spacers and reported an average TTR of 66 days for all patients (mean ± Standard Deviation: 9.4 ± 3.5 weeks) (27). Fifteen patients had a reinfection and their average TTR was 71 days (10.2 ± 4.0 weeks) compared to 64 days (9.2 ± 4.0 weeks) in uninfected patients, however this difference was not significant (*p* = 0.393). The lack of statistical validation may be due to the considerably small number of patients.

Tan et al. investigated the association of the antibiotic holiday with the risk for reinfection after two-stage revision in a large retrospective cohort of 409 patients in two institutions over 14 years from 2000 to 2014 (28). All patients that had additional surgery in the interim period between the stages were excluded. No association with the duration of the antibiotic holiday was found, but with TTR. When graphed alongside the treatment failure rate a steep increase of the treatment failure rate was observed after 100 days TTR. The average TTR for patients without treatment failure in their study was reported 87.9 days and 112.8 days for patients with treatment failure (*p* = 0.037).

Sigmund et al. in 2019 defined ten weeks as a cutoff between a short and a long TTR interval in a retrospective cohort of 93 patients with hip PJI (29). The infection free survival after one year amounted to 94% for the group with the short interval and 91% in the long interval group. At 24 months the survival was 94% (short interval) and 86% (long interval). However, these differences were not significantly different (log-rank test, *p* = 0.223), potentially due to the small number of only nine patients with observed reinfections.

Vielgut et al*.* analyzed 76 patients with acute and chronic hip PJI that were treated with two-stage exchange arthroplasty from 2005 to 2010 (30). Most patients in their cohort received spacers that consisted of a femoral stem with metal head, wrapped in antibiotic-loaded cement. Reimplantation of a prosthesis was planned once the infection was considered eradicated. This required a regular clinical and laboratory examination, three negative joint aspirates and a normal leukocyte scintigraphy. Intraoperative frozen sections and local status at the second stage determined, whether an endoprosthesis was reimplanted or the spacer was exchanged. Thirteen cases required spacer exchange. On average TTR amounted to 12.6 weeks. A TTR-threshold was calculated using the maximally selected log-rank statistic by Hothorn and Lausen (37). This method calculates a cutoff where the survival data yields the biggest difference between two groups. A significantly higher reinfection rate was observed when TTR was less than four weeks or more than eleven weeks. The authors concluded that the optimal TTR, therefore, lies within this timeframe. However, the <4 weeks group contained only five patients, that were all reinfected during the observation period, thus limiting validity. In addition, eight patients that were not fit for second stage surgery due to other preconditions and thirteen patients that required spacer exchange were not excluded from the analysis. Therefore, the authors conclude that the association of TTR with reinfection might be biased by worse overall health condition in the group with longer TTR.

A more recent publication of the same group from 2021 analyzed 77 patients with knee PJI (31). Using a similar methodology, they calculated an optimal cutoff of 83 days (11.8 weeks) for this cohort. The risk for reinfection after the second stage was increased sixfold for patients with a longer interval. In contrast to the patient cohort with hip PJI no second cutoff was identified. Again, patients with spacer exchanges in the interval period were not excluded and no adjustment for the host status was performed, although both factors were identified as significant predictors for reinfection.

In 2022 Borsinger et al*.* reported an increased rate of reinfection after two years for patients with TTR of more than 18 weeks [Odds ratio, CI 95%: 4.12 (1.18–15.37)] (32). Adjustment for comorbidities and previous revision surgeries was done in a cohort of 90 patients with hip and knee PJI (after excluding eleven patients with spacer exchange or Girdlestone resection arthroplasty in the spacer interval). Another group (TTR: 12–18 weeks) had higher odds of treatment failure compared to a group with TTR <12 weeks (odds ratio, CI 95%: 1.89 (0.67–5.77), although not significantly different. The cutoffs at 12 and 18 weeks were defined arbitrarily resulting in groups of similar group size. The calculation of an optimal cutoff with the method by Hothorn and Lausen (37) and additionally a consideration of TTR as a continuous variable would have been interesting. The patient cohort was heterogenous as hip and knee PJI was reported together and the type of knee spacer was inconsistent (static and mobile, prefabricated and handmade, some containing polyethylene tibial components in the PMMA).

Hartman et al. in 2022 reported on a retrospective cohort of 158 patients with hip and knee PJI that underwent both stages with mainly articulating spacers (33). The overall reinfection rate was reported as 19.6% (31/158) and the median TTR in the group with reinfection was 141 days compared to 109 days in the group without reinfection, although not statistically significant (*p* = 0.055). No information on potential revision surgeries between stages was reported.

## Discussion

Few studies have systematically analyzed the potential association of outcomes with TTR in the concept of two-stage revision arthroplasty. However, this topic has recently received increasing attention. This is reflected by the fact that seven of the included eleven studies were published after 2019. The identified studies showed that shorter intervals can achieve comparable or even better infection control compared to longer TTR. In Borsinger's study, this difference was still significant even after adjustment for potential confounding factors and exclusion of all patients with additional surgeries in the interim phase (32).

In chronic PJI, pathogens had long time to penetrate deep into tissue and form mature biofilms on surface areas. Recent findings have shown that *S. aureus* is able to invade deep into the bone *via* the osteocyte lacuno-canalicular network (38). This highlights the need for a radical debridement during the first stage in order to reduce the bacterial load. However, it is difficult to clearly identify infiltrated bone and define “clean” resection margins (39). In the concept of the two-stage exchange arthroplasty, any remaining bacteria after the first stage should be completely eradicated by antibiotic therapy. In addition to systemic therapy, the use of local antibiotics is well established. In many cases antibiotic loaded temporary cement spacers are a preferred treatment concept for chronic PJI (7, 8). The spacer has the task of filling the dead space, stabilizing the joint, maintaining the length of the extremity and releasing local anti-infective substances. Nevertheless, elution decreases over time and the amount of this decrease depends on various factors such as surface size, dosage, mixing technique and choice of antibiotic among other factors (40–43). Without relevant antibiotic elution the spacer acts as a foreign body that could be recolonized by remaining bacteria as observed after sonication of retrieved spacers (21, 44, 45). To avoid this situation, it seems reasonable to keep TTR as short as possible. Additional modern drug delivery systems are commercially available, such as calcium sulfate, that can deliver antibiotics over the time the carrier substance is resorbed (46). Other drug delivery systems such as anti-infective microspheres with high bone affinity are currently being investigated (47).

Another possible explanatory approach for the phenomenon of increased risk of reinfection after long TTR could be the following. The interim phase before reimplantation often means immobilization for elderly patients, especially if static spacers are used and weight bearing is not recommended. Immobilization promotes major complications, including pressure ulcers, pneumonia, urinary tract infection and thromboembolic events (48). Besides a significant reduction of muscle mass in elderly patients (49), negative effects of bed rest are also observed for the immune system (50, 51). It therefore seems plausible that patients with a deteriorated immune system after long immobilization periods could be more prone to reinfection.

These considerations suggest that there is a strong case for shorter spacer intervals. Following this line of reasoning, one could question the value of the two-stage exchange compared to the increasingly propagated one-stage exchange (52, 53). However, It has become accepted that certain conditions are regarded as contraindication for the one-step exchange, such as severe immunocompromise, significant soft-tissue or bony compromise and acute sepsis (54). Therefore, a certain minimum duration of TTR seems justified, but it is still not clear whether this is in the range of 2–4 weeks or longer. The optimal TTR probably depends on various patient specific factors. This circumstance demands a great deal of experience from the surgeons, which confirms that septic revision arthroplasty should be performed at specialised centres with a high caseload.

The question arises why, in clinical trials with large patient cohorts, the reported TTR has so far been significantly longer than known guidelines recommend (4, 7, 14–22). An important factor currently preventing the introduction of short spacer intervals seems to be rules in hospital payment systems (55–57). Many countries, including the United States, Germany and the United Kingdom, have introduced rules that make another surgery for the same diagnosis financially unattractive within a certain period after discharge, which is usually 30 days (58, 59). These measures, which were supposed to improve quality of care by penalizing inappropriately early discharges, have the potential of nudging surgeons to schedule second stage reimplantation later. The consideration of the second stage as a separate case becomes evident in economic analyses, where the second stage reimplantation is classified as an “aseptic” revision case (56, 57). This leads to the situation that the second stage competes for scarce capacity with other surgeries considered “elective”. In the context of a general shortage of hospital capacity, aggravated by the Covid-19 pandemic it is to be expected that implementing shorter TTR will become even more difficult (60, 61). The potential future increase in waiting times for the second stage reimplantation should be closely monitored in registries. The interpretation of the second stage reimplantation as an “aseptic” elective revision case appears inappropriate and should rather be considered as “ongoing infection treatment” that ends only after the antibiotics have been completed after reimplantation. A reasonable consideration to address this barrier seems to be for insurers and health policy makers to provide financial incentives for reimplantation to occur during one inpatient stay or shortly thereafter, as this could reduce the societal costs associated with long-term immobilized patients (62) and could achieve, at least, similar infection control.

This systematic review has substantial limitations. Thus, the results should be interpreted with caution. The most important limitation is the compromised comparability of the studies due to different study designs, small sample size, different definition of treatment success and statistical approaches. Most studies did not evaluate TTR as the primary outcome. Rather, it was one parameter among many to identify potential risk factors as part of an exploratory approach. Although the studies report a measure of the overall health status of patients, it is certainly possible that other factors that were not considered in most studies, such as the virulence of microorganisms, soft tissue condition, nutritional status, wound healing, treatment adherence, or other patient-specific factors, had a relevant impact on TTR and infection control. Spacer exchanges or wound revisions in the interim period prolonged the TTR and this is considered a risk factor for reinfection. But most studies did not exclude these cases. In addition, patients who a surgeon believes might be at a higher likelihood of treatment failure based on clinical experience may have been monitored longer before reimplantation in order to detect persisting infection or reinfection. Only the study by Winkler et al*.* included patients in two different time periods, quasi randomized, to longer or shorter TTR, however the cohort of 38 patients was small and reinfection was observed only once in the whole cohort (14). Because of this variety, a meta-analysis of the results is currently not possible. We suggest for future studies to exclude all patients that require surgery between the stages and to perform adequate adjustment for confounding factors.

## Conclusion

The optimal time to reimplantation within the concept of two-stage revision arthroplasty is not yet defined conclusively. Current evidence suggests that short time to reimplantation might be associated with similar or even better infection control compared to long intervals, although cohorts in the existing literature are still rather small and inhomogeneous. This hypothesis should be investigated in larger clinical studies with standardized outcome parameters and adequate adjustment for potential confounding factors.
