# Supplementary material for: Is long time to reimplantation a risk factor for reinfection in two-stage revision for periprosthetic infection? A systematic review of the literature
Source: Front Surg. 2023 Feb 17;10:1113006. doi: 10.3389/fsurg.2023.1113006 (PMC9981955; doi:10.3389/fsurg.2023.1113006)
Supplement: Supplementary file 1 [file Table1.docx]

# Supplementary Material

# Search Algorithm

# Pubmed:

## Concept 1: Arthroplasty

"Prostheses and Implants"[Mesh] OR "Arthroplasty"[Mesh]) OR "Arthroplasty, Replacement, Knee"[Mesh] OR "Arthroplasty, Replacement, Hip"[Mesh] OR "Arthroplasty, Replacement"[Mesh] OR "Hemiarthroplasty"[Mesh] OR "Bone-Anchored Prosthesis"[Mesh] OR "Knee Prosthesis"[Mesh] OR "Hip Prosthesis"[Mesh] OR "Joint Prosthesis"[Mesh] OR Joint Replacement[tiab]

## Concept 2: Infection

“infections"[Mesh] OR “persistent infection"[Mesh] OR "Bacterial Infections"[Mesh] OR infect*[tiab] OR “surgical wound infection”[Mesh] OR surgical site infection[tiab] OR prostethic joint infection[tiab] OR periprosthetic joint infection[tiab] OR orthopaedic device-related infection[tiab] OR SSI[tiab] OR PJI[tiab] OR ODRI[tiab]

## Concept 3: treatment

"Debridement"[Mesh] OR debridement*[tiab] OR excision*[tiab] OR exchange[tiab] OR "Device Removal"[Mesh] OR removal*[tiab] OR "Anti-Infective Agents"[Mesh] OR "Anti-Bacterial Agents"[Mesh] OR anti-infective*[tiab] OR antiinfective*[tiab] OR antiseptic*[tiab] OR microbicid*[tiab] OR antimicrobial[tiab] OR anti-microbial[tiab] OR antibiotic*[tiab] OR antibacterial[tiab] OR antibacterial[tiab] OR bacteriocid*[tiab] OR Girdlestone*[tiab] OR Resection[tiab] OR Spacer[tiab] OR two-stage*[tiab] OR revision[tiab] OR reimplantation[tiab] OR timing[tiab] OR time[tiab] OR interval[tiab] OR spacer[tiab] OR interstage[tiab] OR period[tiab]

## Concept 4: humans

"Humans"[Mesh] OR human*[tiab] OR "Patients"[Mesh] OR patient[tiab] OR patients[tiab]

## Concept 5: exclusion of case reports and reviews

"Case Reports" [Publication Type] OR case report*[tiab] OR case stud*[tiab] OR case series[tiab] OR case histor*[tiab] OR "Review" [Publication Type] OR review[tiab] OR technical[tiab] OR note[tiab]

## Complete algorithm: **3902** Results on 03/01/2023 23:12:30:

("Prostheses and Implants"[Mesh] OR "Arthroplasty"[Mesh]) OR "Arthroplasty, Replacement, Knee"[Mesh] OR "Arthroplasty, Replacement, Hip"[Mesh] OR "Arthroplasty, Replacement"[Mesh] OR "Hemiarthroplasty"[Mesh] OR "Bone-Anchored Prosthesis"[Mesh] OR "Knee Prosthesis"[Mesh] OR "Hip Prosthesis"[Mesh] OR "Joint Prosthesis"[Mesh] OR Joint Replacement[tiab]) AND ("infections"[Mesh] OR "persistent infection"[Mesh] OR "Bacterial Infections"[Mesh] OR infect*[tiab] OR "surgical wound infection"[Mesh] OR surgical site infection[tiab] OR prostethic joint infection[tiab] OR periprosthetic joint infection[tiab] OR orthopaedic device-related infection[tiab] OR SSI[tiab] OR PJI[tiab] OR ODRI[tiab]) AND ("Debridement"[Mesh] OR debridement*[tiab] OR excision*[tiab] OR exchange[tiab] OR "Device Removal"[Mesh] OR removal*[tiab] OR "Anti-Infective Agents"[Mesh] OR "Anti-Bacterial Agents"[Mesh] OR anti-infective*[tiab] OR antiinfective*[tiab] OR antiseptic*[tiab] OR microbicid*[tiab] OR antimicrobial[tiab] OR anti-microbial[tiab] OR antibiotic*[tiab] OR antibacterial[tiab] OR antibacterial[tiab] OR bacteriocid*[tiab] OR Girdlestone*[tiab] OR Resection[tiab] OR Spacer[tiab] OR two-stage*[tiab] OR revision[tiab] OR reimplantation[tiab] OR timing[tiab] OR time[tiab] OR interval[tiab] OR spacer[tiab] OR interstage[tiab] OR period[tiab]) AND ("Humans"[Mesh] OR human*[tiab] OR "Patients"[Mesh] OR patient[tiab] OR patients[tiab]) NOT ("Case Reports" [Publication Type] OR case report*[tiab] OR case stud*[tiab] OR case series[tiab] OR case histor*[tiab] OR "Review" [Publication Type] OR review[tiab] OR technical[tiab] OR note[tiab])

NCBI filters set: time: 1970-2023; Text availability: Full text; Article type: Clinical Study, Clinical Trial, Comparative Study, Controlled Clinical Trial, Multicenter Study, Observational Study, Pragmatic Clinical Trial, Ranomized Controlled Trial

# Cochrane Library:

## Concept 1: Arthroplasty

(Arthroplasty):ti,ab,kw OR ("prosthesis"):ti,ab,kw OR ("knee replacement"):ti,ab,kw OR ("hip replacement arthroplasty"):ti,ab,kw OR ("hemiarthroplasty"):ti,ab,kw OR (joint replacement):ti,ab,kw OR (knee *prosthesis):ti,ab,kw OR (hip *prosthesis):ti,ab,kw OR (joint *prosthesis):ti,ab,kw OR (endoprosthesis):ti,ab,kw

## Concept 2: Infection

("bacterial infection") OR (infection*):ti,ab,kw OR ("surgical wound infection"):ti,ab,kw OR ("surgical-site infection"):ti,ab,kw OR (*prosthetic join infection):ti,ab,kw OR (biofilm) OR (implant-related infection):ti,ab,kw OR (orthop* device-related infection) :ti,ab,kw OR (ODRI):ti,ab,kw OR (SSI):ti,ab,kw OR (PJI):ti,ab,kw

## Concept 3: treatment

("débridement"):ti,ab,kw OR (debridement):ti,ab,kw OR (excision):ti,ab,kw OR (revision arthroplasty):ti,ab,kw OR (two-stage revision):ti,ab,kw OR ("Girdlestone procedure"):ti,ab,kw OR ("Girdlestone situation"):ti,ab,kw OR (resection arthroplasty):ti,ab,kw OR (implant removal):ti,ab,kw OR (exchange):ti,ab,kw OR ("antiinfectives"):ti,ab,kw OR ("antibacterial"):ti,ab,kw OR ("antibiotic drug"):ti,ab,kw OR ("bactericidal"):ti,ab,kw OR ("antimicrobial"):ti,ab,kw

## Concept 4: humans

(humans) OR (human*):ti,ab,kw OR (patient*):ti,ab,kw

## Concept 5: exclusion of case reports and reviews

(case series) OR (review*):ti,ab,kw OR (case report):ti,ab,kw OR (case report*):ti,ab,kw OR (case study):ti,ab,kw OR ("case-history"):ti,ab,kw

## Complete algorithm: **489** Results on 03/01/2023 22:01:34:

((Arthroplasty):ti,ab,kw OR ("prosthesis"):ti,ab,kw OR ("knee replacement"):ti,ab,kw OR ("hip replacement arthroplasty"):ti,ab,kw OR ("hemiarthroplasty"):ti,ab,kw OR (joint replacement):ti,ab,kw OR (knee *prosthesis):ti,ab,kw OR (hip *prosthesis):ti,ab,kw OR (joint *prosthesis):ti,ab,kw OR (endoprosthesis):ti,ab,kw) AND (("bacterial infection") OR (infection*):ti,ab,kw OR ("surgical wound infection"):ti,ab,kw OR ("surgical-site infection"):ti,ab,kw OR (*prosthetic join infection):ti,ab,kw OR (biofilm) OR (implant-related infection):ti,ab,kw OR (orthop* device-related infection) :ti,ab,kw OR (ODRI):ti,ab,kw OR (SSI):ti,ab,kw OR (PJI):ti,ab,kw) AND (("débridement"):ti,ab,kw OR (debridement):ti,ab,kw OR (excision):ti,ab,kw OR (revision arthroplasty):ti,ab,kw OR (two-stage revision):ti,ab,kw OR ("Girdlestone procedure"):ti,ab,kw OR ("Girdlestone situation"):ti,ab,kw OR (resection arthroplasty):ti,ab,kw OR (implant removal):ti,ab,kw OR (exchange):ti,ab,kw OR ("antiinfectives"):ti,ab,kw OR ("antibacterial"):ti,ab,kw OR ("antibiotic drug"):ti,ab,kw OR ("bactericidal"):ti,ab,kw OR ("antimicrobial"):ti,ab,kw) AND ((humans) OR (human*):ti,ab,kw OR (patient*):ti,ab,kw) NOT ((case series) OR (review*):ti,ab,kw OR (case report):ti,ab,kw OR (case report*):ti,ab,kw OR (case study):ti,ab,kw OR ("case-history"):ti,ab,kw)

# Web of Science (WoS)

## Concept 1: Arthroplasty

ALL=(Arthroplasty) OR ALL=(prosthesis) OR ALL=(knee replacement) OR ALL=(hip replacement) OR ALL=(hemiarthroplasty) OR ALL=(joint replacement) OR ALL=(knee prosthesis) OR ALL=(hip prosthesis) OR ALL=(joint prosthesis) OR ALL=(endoprosthesis)

## Concept 2: Infection

ALL=(bacterial infection) OR ALL=(infection*) OR ALL=(surgical wound infection) OR ALL=(surgical site infection) OR ALL=(prosthetic joint infection) OR ALL=(biofilm) OR ALL=(implant-related infection) OR ALL=(orthop* device-related infection) OR ALL=(ODRI) OR ALL=(SSI) OR ALL=(PJI)

## Concept 3: treatment

ALL=(debridement) OR ALL=(spacer) OR ALL=(excision) OR ALL=(revision arthroplasty) OR ALL=(two-stage revision) OR ALL=(Girdlestone) OR ALL=(resection arthroplasty) OR ALL=(implant removal) OR ALL=(exchange) OR ALL=(aniinfectives) OR ALL=(antibacterial) OR ALL=(antibiotic) OR ALL=(bactericidal) OR ALL=(antimicrobial) OR ALL=(reimplantation)

## Concept 4: humans

ALL=(human*) OR ALL=(patient*)

## Concept 5: exclusion of case reports and reviews

ALL=(case series) OR ALL=(review*) OR ALL=(case report) OR ALL=(case study) OR ALL=(case history)

## Complete algorithm: **1619** results on 04/01/2023 20:01:21:

((ALL=(Arthroplasty) OR ALL=(prosthesis) OR ALL=(knee replacement) OR ALL=(hip replacement) OR ALL=(hemiarthroplasty) OR ALL=(joint replacement) OR ALL=(knee prosthesis) OR ALL=(hip prosthesis) OR ALL=(joint prosthesis) OR ALL=(endoprosthesis)) AND (ALL=(bacterial infection) OR ALL=(infection*) OR ALL=(surgical wound infection) OR ALL=(surgical site infection) OR ALL=(prosthetic joint infection) OR ALL=(biofilm) OR ALL=(implant-related infection) OR ALL=(orthop* device-related infection) OR ALL=(ODRI) OR ALL=(SSI) OR ALL=(PJI)) AND (ALL=(debridement) OR ALL=(spacer) OR ALL=(excision) OR ALL=(revision arthroplasty) OR ALL=(two-stage revision) OR ALL=(Girdlestone) OR ALL=(resection arthroplasty) OR ALL=(implant removal) OR ALL=(exchange) OR ALL=(aniinfectives) OR ALL=(antibacterial) OR ALL=(antibiotic) OR ALL=(bactericidal) OR ALL=(antimicrobial) OR ALL=(reimplantation) OR ALL=(interval) OR ALL=(time) OR ALL=(duration) OR ALL=(interim) OR ALL=(period) OR ALL=(interstage) OR ALL=(timing) AND (ALL=(human*) OR ALL=(patient*)) NOT (ALL=(case series) OR ALL=(review*) OR ALL=(case report) OR ALL=(case study) OR ALL=(case history))))

Refined by: citation topics meso: Orthopedics and Antimicrobials & Antimicrobials
